# Understanding the Interplay between Expression, Mutation and Activity of ALK Receptor in Rhabdomyosarcoma Cells for Clinical Application of Small-Molecule Inhibitors

**DOI:** 10.1371/journal.pone.0132330

**Published:** 2015-07-06

**Authors:** Marica Peron, Federica Lovisa, Elena Poli, Giuseppe Basso, Paolo Bonvini

**Affiliations:** 1 Clinica di Oncoematologia Pediatrica di Padova, Azienda Ospedaliera-Università di Padova, Padua, Italy; 2 Istituto di Ricerca Pediatrica Città della Speranza, Padova, Italy; Thomas Jefferson University, UNITED STATES

## Abstract

**Background:**

Receptor tyrosine kinases (RTKs) have a central role in cancer initiation and progression, since changes in their expression and activity potentially results in cell transformation. This concept is essential from a therapeutic standpoint, as clinical evidence indicates that tumours carrying deregulated RTKs are particularly susceptible to their activity but also to their inhibition. Rhabdomyosarcoma (RMS) is an aggressive childhood cancer where emerging therapies rely on the use kinase inhibitors, and among druggable kinases ALK represents a potential therapeutic target to commit efforts against. However, the functional relevance of ALK in RMS is not known, likewise the multi-component deregulated RTK profile to which ALK belongs.

**Methods:**

In this study we used RMS cell lines representative of the alveolar and embrional histotype and looked at ALK intracellular localization, activity and cell signalling.

**Results:**

We found that ALK was properly located at the plasma membrane of RMS cells, though in an unphosphorylated and inactive state due to intracellular tyrosine phosphatases (PTPases) activity. Indeed, increase of ALK phosphorylation was observed upon PTPase inhibition, as well as after ligand binding or protein overexpression. In these conditions, ALK signalling proceeded through the MAPK/ERK and PI3K/AKT pathways, and it was susceptible to ATP-competitive inhibitors exposure. However, drug-induced growth inhibition, cell cycle arrest and apoptosis did not correlate with ALK expression only, but relied also on the expression of other RTKs with akin drug binding affinity. Indeed, analysis of baseline and inducible RTK phosphorylation confirmed that RMS cells were susceptible to ALK kinase inhibitors even in the absence of the primary intended target, due to the presence of compensatory RTKs signalling pathways.

**Conclusions:**

These data, hence, provided evidences of a potentially active role of ALK in RMS cells, but also suggest caution in considering ALK a major therapeutic target in this malignancy, particularly if expression and activity cannot be accurately determined.

## Introduction

Anticancer drug development attempts to translate knowledge gained from basic research into clinical trials using approaches selectively targeting oncogenic molecules in cancer cells. In this context, protein kinases have emerged as a novel focus of current anticancer research, since most of them act in oncogenic pathways in a rate-limiting manner, rendering tumour cells addicted to their unusual high expression and activity [1,2]. As such, the identification of a druggable oncogenic kinase represents nowadays an important therapeutic upshot across several malignancies, and targeting single or multiple protein kinase pathways has been recently exploited with positive results in advanced cancers refractory to standard chemotherapy [3,4,5,6]. In cancer cells, however, kinase inhibitor efficacy may be limited by several resistance mechanisms, including secondary mutations in the primary oncogenic kinase (intrinsic resistance) or redundant signalling pathways activated upon targeted inhibition (acquired resistance) [7,8,9]. Clinical approaches to overcome such a drug resistance rely, thus, on the development of novel inhibitors with increased potency and selectivity, but also on compounds capable of targeting compensatory signalling pathways that may switch cancer cells to a new dependency [10,11,12,13].

Rhabdomyosarcoma (RMS) is a soft tissue sarcoma of the childhood characterized by the aberrant expression of multiple receptor tyrosine kinases (RTK), and great effort has been devoted to understand whether activated RTKs may represent promises for the development of new drugs and therapies. In RMS, major characteristic of tumour aggressiveness is represented by the PAX3/7-FOXO1 fusion gene, a negative prognostic factor that regulates transcription of several downstream tumour-driving genes, including c-Met, CXCR4, FGFR4, IGFR-1R and PDGFRα kinases [14,15,16]. However, high RTK expression correlates with inferior outcome in PAX3/7-FOXO1-negative tumours as well, suggesting that this class of proteins likely contributes to the growth and survival of this malignancy [17,18,19,20]. Nevertheless, while compelling evidences of RTK oncogenic activity *in vitro* have been reported, their value as therapeutic targets *in vivo* remains uncertain, given that inhibition of a single protein kinase does not appear sufficient treatment for cancer like RMS in which functional RTK redundancy is observed [21,22,23,24].

We and others have recently shown that anaplastic lymphoma kinase (ALK) is another transmembrane receptor that identifies high-risk tumours independently of PAX3-FOXO1 expression and RMS histology [25,26,27]. However, whether ALK has a role in RMS initiation and maintenance, or it may represent a novel therapeutic target, remains to be ascertained. ALK is a RTK that can be implicated as truncated fusion protein and full-length kinase in a number of solid and haematologic malignancies, and in most cases its inhibition leads to a marked decrease of tumour cells growth and survival [28,29,30,31,32]. Nowadays, ALK small-molecule inhibitors are among the most promising agents in several high-risk cancers, based on the fact that ALK, when activated by mutation, amplification or gene rearrangement, becomes highly oncogenic [33,34,35,36]. However, early clinical reports indicate that patients respond favourably to selective inhibitors if a near complete inhibition of ALK kinase activity is achieved and protein levels are sufficiently high to sustain its continuous activation [37,38].

Herein, we assessed ALK expression and function in RMS cells, *in vitro*, and demonstrated that full-length ALK receptor localizes at the plasma membrane but lacks of constitutive activity due to permanent dephosphorylation by endogenous tyrosine phosphatases (PTPases). Nonetheless, we provided evidence that protein overexpression or ligand-induced receptor dimerization promoted ALK activation and signalling in these cells, whereas drug treatment resulted in cell cycle arrest and apoptosis. However, we also found that well-known ALK inhibitor compounds were active in ALK-negative RMS cells, as the result of compensatory RTKs inhibition. Taken together, these findings provide a better understanding of the state of ALK in RMS cells, but also raise questions about its druggability *in vivo*.

## Materials and Methods

### Cell culture and treatment

The human rhabdomyosarcoma (RMS) cell lines RH30 and RD were purchased from ATCC (Manassas, VA), whereas RH4 cells were a gift of Dr P.J. Houghton (St Jude Children’s Hospital, Memphis, TN). The neuroblastoma (NB) cell lines NB1 and SH-SY5Y were obtained from Dr GP. Tonini and L. Longo (IRCCS San Martino Hospital, Genova, Italy), whereas SU-DHL1 anaplastic large cell lymphoma cells (ALCL) were purchased from ATCC (Manassas, VA). HEK-293T cells were a generous gift of Dr. S. Indraccolo (Istituto Oncologico Veneto, IOV, Padova, Italy). RMS, NB and HEK-293T cells were all grown in Dulbecco’s Modified Eagle’s Medium supplemented with 10% heat-inactivated foetal calf serum (FCS) (Gibco, Life Technologies Co., Carlsbad, CA, USA), whereas SU-DHL-1 cells were maintained in RPMI 1640 with 15% FCS. All the cell lines were characterized for ALK (RH30, RH4, NB1 and SH-SY5Y), NPM-ALK (SU-DHL-1), or PAX3-FOXO1 expression.

### Reagents and antibodies

ALK small-molecule inhibitors PF-02341066 (Crizotinib) and NVP-TAE684 were purchased from Selleckchem (Selleck Chemicals, Houston, TX, USA), whereas anti-ALK monoclonal antibodies (mAb46 and 30) were a generous gift of Dr. Marc Vigny [39]. The primary antibodies for Western blot analysis were from Cell Signalling (ALK^Y1604^; AKT^S473^; ERK^T202/Y204^; c-Met^Y1234/1235^; EGFR^Y1068^; IGF1-Rβ^Y1135/1136^; IGF1-Rβ) (Cell Signaling Technology, Inc., Danvers, MA, USA); SIGMA (γ-tubulin) (SIGMA Aldrivh Co., USA); Invitrogen (ALK) (Invitrogen, Life Technologies Co); or Santa Cruz (AKT; ERK) (Santa Cruz Biotechnology, Inc., Santa Cruz, CA, USA). Horseradish peroxidase (HRP)-conjugated sheep anti-mouse or donkey anti-rabbit antibodies, were purchased from GE Healthcare (GE Healthcare Life Sciences, Uppsala, Sweden), whereas recombinant human HGF, EGF, and IGF-1 growth factors were purchased from Peprotech (Peprotech, NJ, USA). Pervanadate stock solution (100mM) was prepared as indicated by Huyer et al. [40]; while MTT salt (3-(4,5-Dimethylthiazol-2-yl)-2,5-diphenyltetrazolium bromide) was available from SIGMA (SIGMA Aldrich Co., USA).

### Cell lysis, immunoblotting and immunoprecipitation

Untreated and treated cells were lysed using standard methods [41]. Briefly, the cells were washed twice in ice-cold PBS and lysed by addition of TritonX-100 sample buffer (50 mM Tris-HCl [pH 7.5]; 130 mM NaCl; 1% Triton X-100; 0.1% SDS; 2 mM EDTA; 1 mM PMSF; 20 μg/ml leupeptin; 20μg/ml aprotinin). The lysates were clarified by high-speed centrifugation and fractionated by SDS-PAGE prior to transferring onto nitrocellulose membranes. Immunoprecipitation was performed by incubating protein lysates (1 mg) with 2 μg of specific antibodies (α-ALK or α-ALK^Y1604^) at 4°C overnight, and the resulting immunocomplexes with 30 μl of Protein G-Sepharose beads for 2 h at 4°C. The immunoadsorbed pellets were washed 4 times with 1% Triton X-100 lysis buffer and heated at 95°C in Laemmli buffer. Aliquots of cell lysates (50–70 μg) and immunoprecipitates were fractionated by SDS-PAGE and transferred to nitrocellulose membranes for Western blot analysis. Protein bands were visualized by chemiluminescence and analyzed by using ImageJ software (National Institute of Health, Bethesda, MD, USA).

### Cell growth and viability

Cell cycle analysis was performed on cells treated for 24 hours, or left untreated, with increasing concentrations of crizotinib or TAE684 inhibitors. The cells were washed in ice-cold PBS, fixed in cold 70% ethanol and resuspended in 0.6 ml PI [50 μg/ml] right before cell cycle analysis with BD FACS-Calibur Cell Cytometer and Macintosh CellQuest software (Becton Dickinson, Italy).

Cell viability was measured by MTT assay every 24 hours up to three days. RMS, NB and ALCL cells were seeded onto 96-well microculture plates 12 hours before drug addition and then grown in the presence or absence of ALK inhibitors as indicated in the text. MTT optical density was measured with Victor3 Multilabel Counter spectrophotometer at 540 nm and values of three independent experiments averaged. Drug-induced growth inhibition of RMS cells in the presence or absence of HGF, IGF-I or EGF growth factors was measured alike.

### Cell surface staining

To assess ALK expression at the plasma membrane, exponentially growing cells were incubated with 10 μg/ml anti-ALK primary monoclonal antibody, washed in PBS, and subsequently labeled with Alexa Fluor 488 conjugated anti-mouse secondary antibody. Staining with primary and fluorochrome-labeled secondary antibodies was performed on ice with ice-cold reagents/solutions, since low temperatures prevent the modulation and internalization of surface antigens that can produce a loss of fluorescence intensity. The cells were kept in the dark, in ice, until fluorescence analysis with BD FACS-Calibur Cell Cytometer (Becton Dickinson, Italy) was carried out. ALK mean fluorescence intensity (MFI) was measured by Macintosh CellQuest software (Becton Dickinson, Italy).

### Constructs and transfection

The full length human ALK cDNA was purchased from ATCC and subcloned into the mammalian expression vector pcDNA3.1. Point mutations F1174L and R1275Q were introduced by site-directed mutagenesis, using the Phusion site-directed mutagenesis kit (Thermo Fisher Scientific Inc., Waltham, MA, USA) and the following oligonucletides: P-TCATCAGCAAATTAAACCACCAGA and P-TCAGGGCTTCCATGAGGAAATC to generate F1174L substitution; P-GGATGGCCCAAGACATCTACA and P-CGAAGTCTCCAATCTTGGCCA to introduce R1275Q mutation. Transfection was then performed using Lipofectamine 2000 reagent (Invitrogen), according to manufacturer’s instructions.

### Data analysis

Results were expressed as mean ± standard error of the mean (s.e.m.) of three independent experiments and analyzed by using the two-sided Student’s t test (SigmaPlot 11.0), with a P value<0.05 considered significant.

## Results

### Plasma membrane localization and activation of ALK kinase in RMS cells

**Fig 1:**
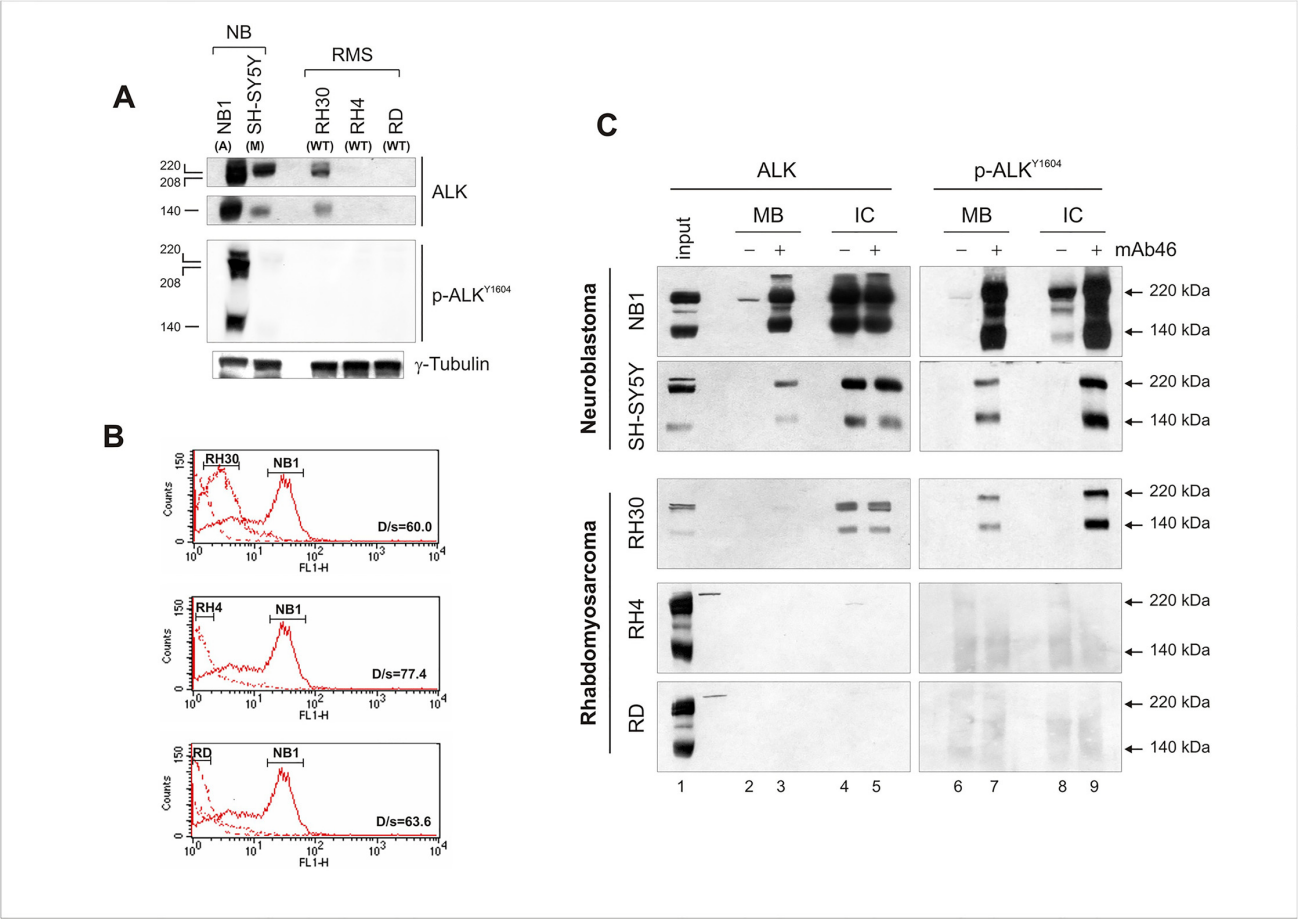
Endogenous ALK expression levels influence receptor intrinsic kinase activity in RMS cells. (A) ALK protein expression (upper panels) and phosphorylation (lower panel) in RMS (RH30, RH4, RD) and neuroblastoma (NB1, SH-SY5Y) cell lines. Western blot analysis of cell extracts using the specified polyclonal anti-ALK (*ALK*) and anti-phospho ALK (*ALK* ^*Y1604*^) antibodies. Full-length (220 kDa) and cleaved ALK form (140 kDa) are shown. *ALK* gene status is also indicated (A = amplified; M = mutated; WT = wild-type) (B) Cell surface detection and quantitative assessment of ALK receptor. Membrane-bound ALK kinase in non-permeabilized RMS and neuroblastoma cells using a primary monoclonal antibody against the N-terminal portion of ALK. Fluorescent-conjugated secondary antibody was used to acquire ALK signal with FACS-Calibur Cell Cytometer. ALK-amplified NB1 cells were included in the analysis as positive control for ALK expression and localization at the plasma membrane. (C) mAb46 effect on ALK receptor activation. RMS (RH30, RH4 and RD) and neuroblastoma (NB1, SHSY-5Y) cells were treated (+) or left untreated (-) with 1 μg/ml agonist mAb46 for 30 min. Membrane-bound (MB) and intracellular (IC) ALK was immunoprecipitated as described in material and methods and detected using polyclonal anti-ALK (*ALK*) and anti-phospho ALK (*ALK* ^*Y1604*^) antibodies. Full-length (220 kDa) and cleaved (140 kDa) ALK proteins position is indicated by arrowheads. NB1 cell extracts were used as positive control for ALK expression in RH4 and RD immunoblots.

To explore baseline expression and phosphorylation of ALK kinase, RMS cell lines representative of the ARMS and ERMS subtype, and previously defined for ALK expression [25], were utilized, together with neuroblastoma cells carrying amplified or mutant (F1174L) *ALK* gene. Consistent with our previous observations, ALK was expressed mainly in PAX3-FOXO1-positive RH30 cells, but lacked of constitutive kinase activity (Fig 1A). Similarly, basal phosphorylation of mutant ALK receptor was very low in SH-SY5Y cells compared to that of NB1 (amplified ALK), suggesting that under some circumstances protein expression overcomes gene mutational status as criteria for intrinsic kinase activity. However, to rule out aberrant protein trafficking and localization, cell surface detection of ALK was carried out using a monoclonal antibody directed toward the extracellular portion of the kinase, in cells kept in ice to avoid antigen modulation and internalization. When compared to ALK-amplified NB1 cells, PAX3-FOXO1-positive RH30 cells showed a weak but detectable ALK expression at the plasma membrane (Fig 1B), whereas in the other two RMS cell lines ALK signal was undistinguishable from background fluorescence. Indeed, when dimerization was induced by exposing cells to agonist mAb46 monoclonal antibody, activation of ALK occurred in RH30 cells, as indicated by the increased degree of phosphorylation of both the plasma membrane and intracellular pool of ALK (Fig 1C, lanes 6–7 and 8–9, respectively) [39,42]. Of note, antibody treatment acted on cell surface-exposed ALK receptor, since phosphorylation of both 220 and 140 kDa ALK (*arrowheads*) was observed, the latter of which corresponds to an extracellular product cleavage of the mature receptor by plasma membrane proteases [43]. These observations were confirmed in NB1 and SH-SY5Y neuroblastoma cells, which were responsive to antibody treatment independently of ALK mutational status (Fig 1, lane 8).

### Consequences of inducible and spontaneous activation of ALK on RMS cell signalling

**Fig 2:**
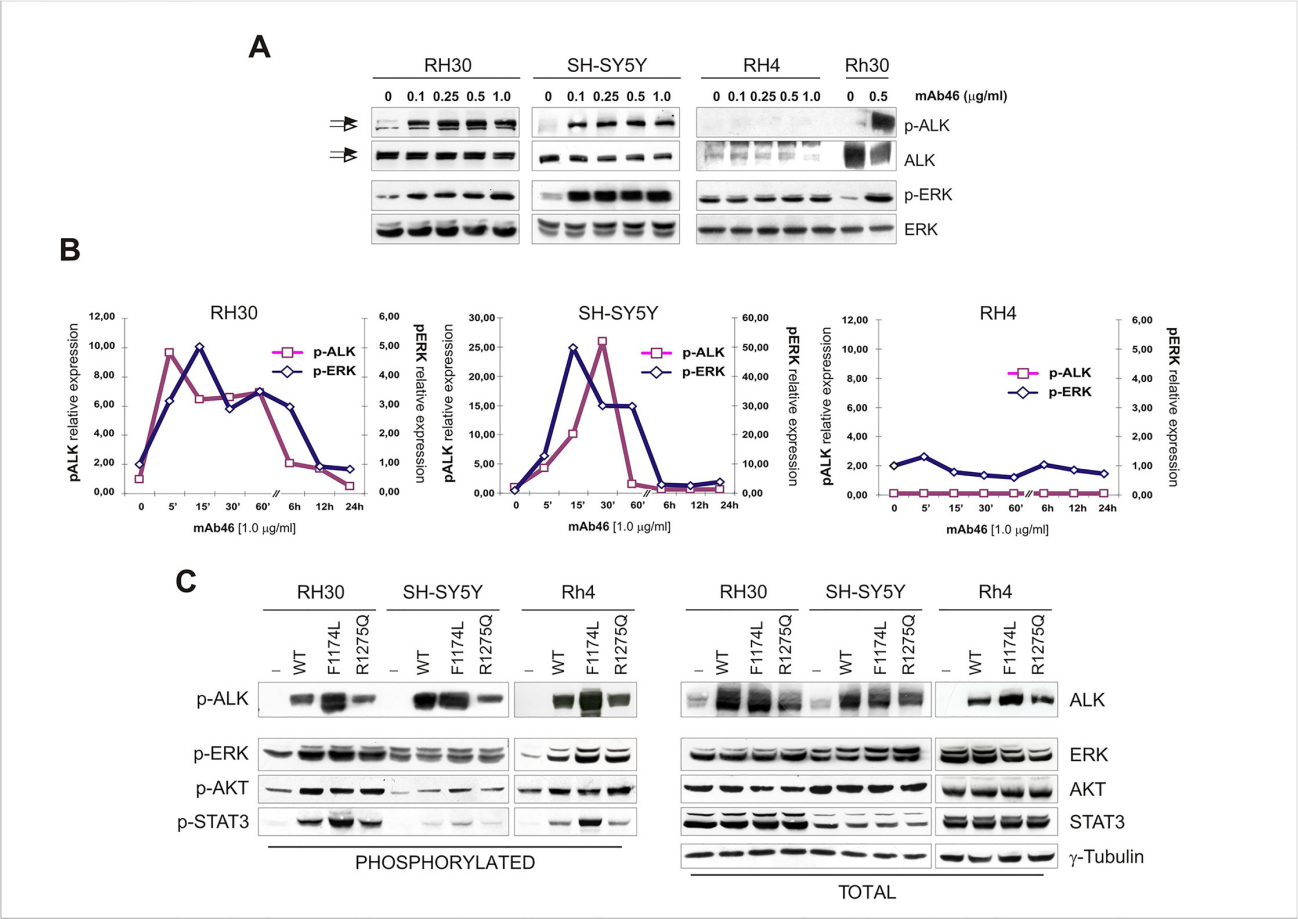
Kinetics of ALK receptor stimulation and signalling. (A) Antibody-dependent ALK activation was induced by exposing RMS (RH30, RH4) and NB (SH-SY5Y) cells to increasing concentration of mAb46 (0.1–1 μg/ml). Cell lysates were subjected to Western blot analysis using polyclonal antibodies for total and phosphorylated ALK or ERK proteins. Arrowheads indicate membrane-bound (*closed*) and cytoplasmic (*open*) full-length ALK kinase, respectively. (**B**) Time-course analysis of ALK phosphorylation after antibody mAb46 treatment. RH30, SH-SY5Y and RH4 cells were exposed to 1 μg/ml mAb46 for increasing time intervals, and ALK phosphorylation was assessed by Western blot analysis at each time point. Phosphorylation of ERK is also shown. Proteins band density was measured and expressed as fold(s) of control in graphs. Phosphorylated ALK and ERK values were graphed using different scale options. (**C**) Ligand-independent ALK receptor activity. Relative expression and phosphorylation of ALK RTK was assessed in RH30, SH-SY5Y and RH4 cells after transfection with wild-type (WT) or mutant (F1174L and R1275Q) ALK expression plasmids. Expression and phosphorylation of ALK, ERK, AKT and STAT3 proteins was assessed, using γ-Tubulin as loading control.

RTK activation is dynamic process that consists of growth factor binding and receptor autophosphorylation before intracellular signal processing [44]. However, recent observations support the concept that self-association of the extracellular regions of RTKs may occur even in the absence of a specific ligand, particularly in cancer cells in which dimerization takes place spontaneously as a consequence of increased protein expression [45,46,47]. Thus, to look more in detail at the mechanisms of ALK activation, RH30 and SH-SY5Y cells were exposed to increasing amounts of agonist mAb46 antibody and kinetics of receptor phosphorylation was assessed. Antibody treatment led to a marked phosphorylation of ALK in both cell lines, which correlated with a prompt and durable activation of ERK kinase (Fig 2A and 2B). In contrast, mAb46 exposure did not have any effect in RH4 cells, consistent with the scarce expression of ALK in these cells. Therefore, to prove also that receptor density at the plasma membrane could affect intrinsic kinase activity, we transiently transfected RH30, SH-SY5Y and RH4 cells with wild-type or mutant receptor constructs, and assessed protein phosphorylation in the absence of ligand binding. Among the previously identified ALK somatic and germline mutations, we choose hot spot residues F1174L and R1275Q, since mutations at these sites account for more than 70% of mutations in neuroblastoma patients and result in a altered receptor activity [33,48]. Consistent with our hypothesis, overexpression of ALK promoted spontaneous receptor dimerization and activity on the membrane and mediated phosphorylation of downstream targets ERK1/2, AKT and STAT3 independently of its mutational status (Fig 2C) [49]. Perhaps the most striking observation from this analysis was that ALK levels affected RMS cell signaling even in the absence of activating mutations or growth factor binding, establishing a novel genotype-therapeutic correlation that can be used to identify patients who most likely respond to ALK kinase inhibitors.

### Putative role of intracellular phosphatases in the regulation of ALK intrinsic kinase activity in RMS cells

**Fig 3:**
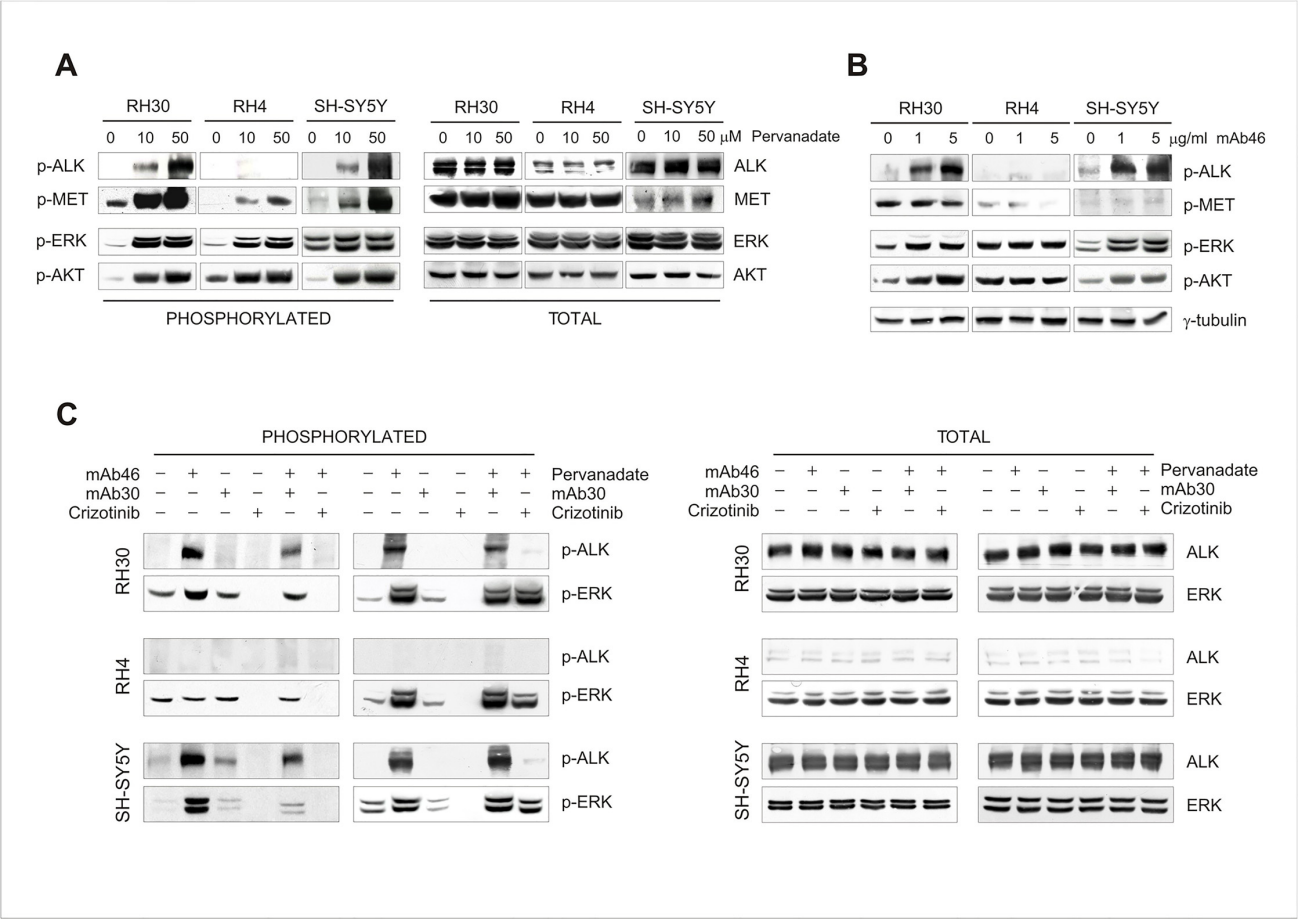
Regulation of ALK kinase activity by intracellular phosphatases. RH30, RH4 and SH-SY-5Y cells were treated with Pervanadate (**A**) or agonist mAb46 monoclonal antibody (**B**). Immunoblottings of total and phosphorylated ALK, c-Met (*MET*), ERK and AKT proteins are shown. (**C**) Regulation of inducible ALK phosphorylation and activity. RH30, RH4 and SH-SY-5Y cells were treated with both mAb46 (3 μg/ml) and Pervanadate (50 μM) to measure ALK phosphorylation and signalling. To inhibit ALK, the cells were pre-incubated with antagonist mAb30 antibody (2 μg/ml) or with Crizotinib inhibitor (1 μM). Protein lysates were subjected to Western blot analysis using antibodies specific for total and phosphorylated ALK and ERK proteins, as described.

The generation of intracellular signalling depends on balanced activities of constitutively active RTKs and protein tyrosine phosphatases (PTPases) [50]. As such, basal activity of weakly expressed RTKs may be not discernable due to permanent dephosphorylation by intracellular PTPases, whereas at high expression levels detection of receptor phosphorylation is feasible [46,47,51]. Thus, to investigate the impact of phosphatases on ALK phosphorylation and activity, pervanadate inhibitor was administered to the cells and the effect compared with that observed after exposure to mAb46. We demonstrated that PTPase inhibition clearly increased ALK phosphorylation in RH30 and SH-SY5Y cells, and led to activation of ERK and AKT target proteins (Fig 3A). However, pharmacological inhibition of intracellular phosphatases by pervanadate promoted a general RTKs activation in these cells, since stimulation of c-Met receptor, as well as phosphorylation of downstream ERK kinase, was observed independently of ALK expression and activity (Fig 3A, RH30 vs. RH4). In contrast, mAb46 treatment selectively activated ALK and had no influence on the activity and signal processing of other RTKs (Fig 3B). Indeed, when ALK antagonist mAb30 antibody or tyrosine kinase inhibitor crizotinib were administered prior to mAb46 or pervanadate exposure, activation of ALK was completely prevented, whereas downregulation of ALK-dependent phosphorylation of ERK was observed only in mAb46-treated cells (Fig 3C).

### Apoptosis induction in RMS cells exposed to ALK small-molecules inhibitors

**Fig 4:**
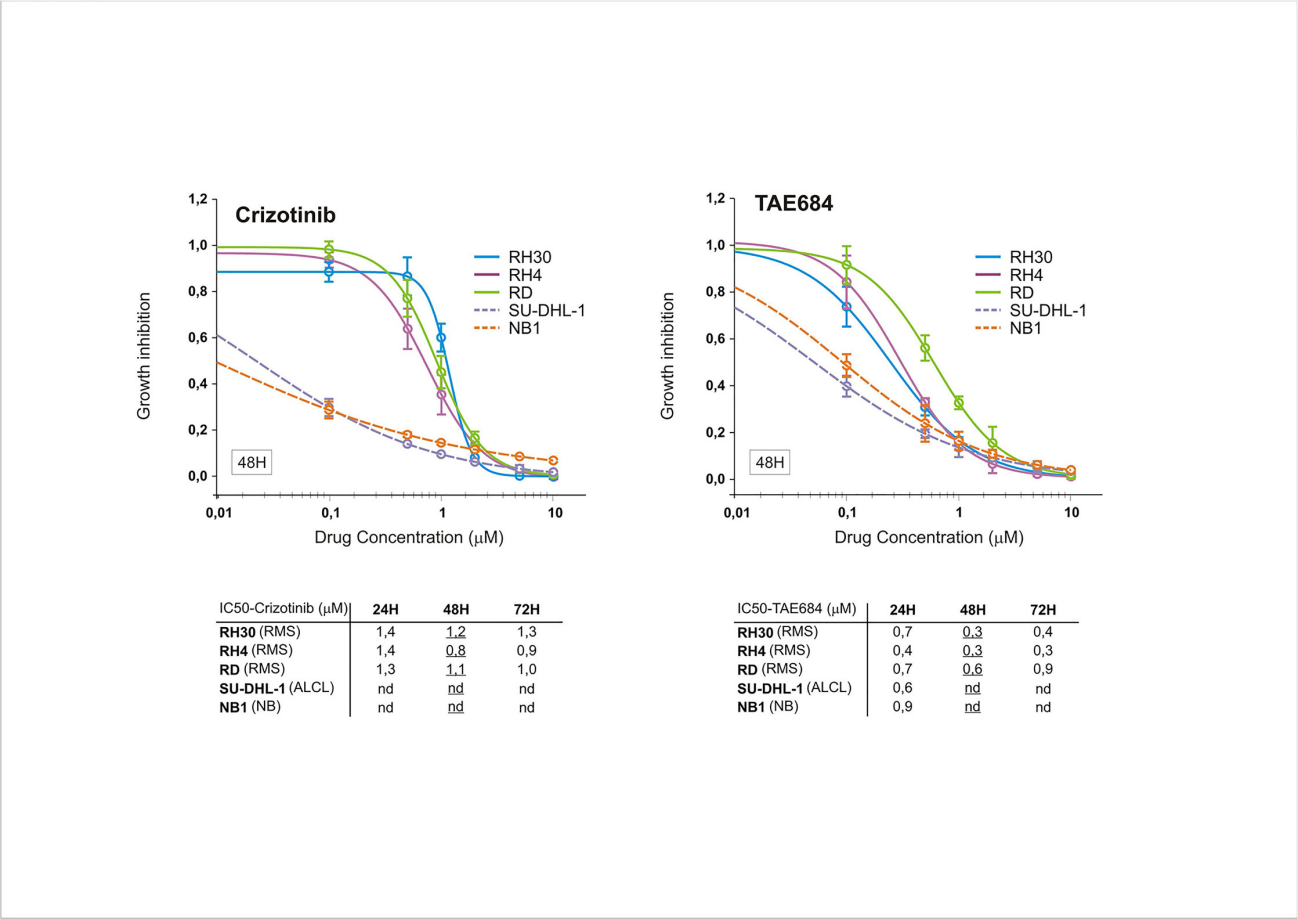
Cytotoxic activity of crizotinib and TAE684 ALK inhibitors. Growth inhibition of RMS cells (RH30, RH4 and RD) treated with the indicated doses of Crizotinib and TAE684 for 48 hours. The cell survival was assayed by MTT assay. Growth inhibition of neuroblastoma (NB1, amplified *ALK*) and ALCL (SU-DHL-1, *NPM-ALK*) cells is also shown. Each time point represents triplicate of biological replicates and it’s representative of three independent experiments. Results are expressed as fold of control ±s.e.m. IC50 values of cell survival assay for Crizotinib and TAE684 calculated every 24 hours up to 72 hours are also reported (below), with 48H-IC50 values underlined.

**Fig 5:**
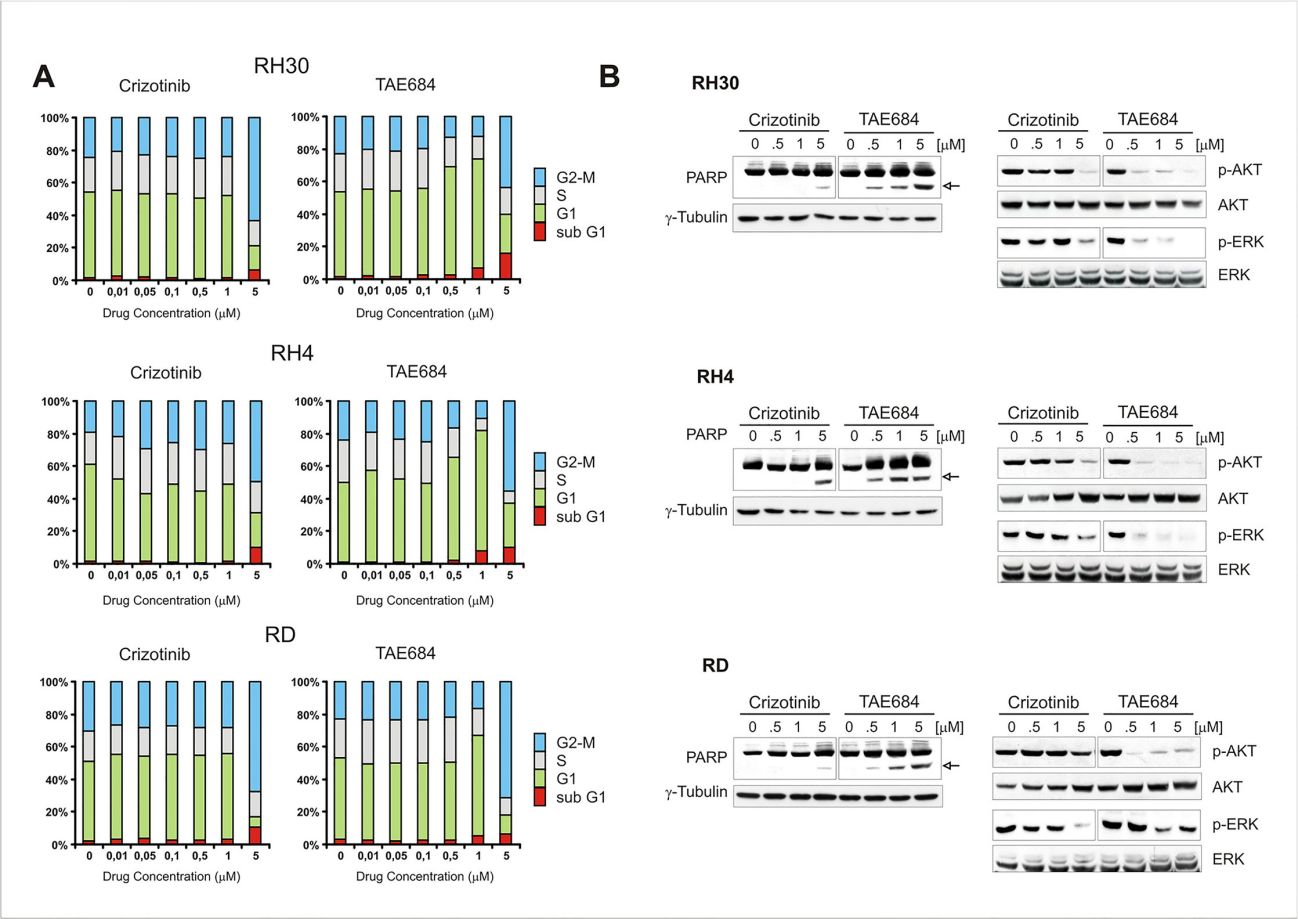
RMS cell growth and signalling is downregulated by ALK inhibitors. (A) Cell cycle analysis of RMS cells treated with the indicated doses of Crizotinib and TAE684 for 24 hours. Cell cycle phases distribution measured with FACS-Calibur Cell Cytometer and represented by stacked bar graphs. The percentage of cells in sub G1 phase is shown. (B) Lysates from ALK inhibitor-treated and untreated RMS cells, probed with antibodies directed against total and phosphorylated AKT and ERK proteins, as well as against full-length and cleaved PARP (*arrowhead*). γ-Tubulin was used as internal loading control.

Almost all protein kinases share the same conserved sequences around the ATP-binding site, as they all have the same phosphotransferase activity toward common or uncommon protein substrates [52]. Small molecules targeting the ATP binding cleft may have, thus, an inherent multi-target nature and be active in cells where the primary target is weakly expressed or even absent [53,54]. Consistent with these findings, we found that crizotinib inhibited basal ERK phosphorylation in RMS cells independently of ALK expression and activity, as this phenomenon was observed in RH4 cells as well (Fig 2C). Therefore, we tested the ability of ALK inhibitors to impede the growth of ALK-positive and–negative RMS cells, when administered at increasing concentrations and prolonged time intervals. Crizotinib was chosen for its dual ALK/Met inhibitor activity, while NPV-TAE684 (TAE684) for its affinity for ALK and, to a lesser extent, IGF-1R [55,56]. We found that at low doses TAE684 showed stronger inhibitory potency than crizotinib, whereas at higher concentrations such difference was less significant and not associated with ALK expression (Fig 4). Conversely, transformed cells overexpressing full-length (NB1) or truncated (SU-DHL-1) ALK kinase displayed a much higher sensitivity compared to RMS cells, consistent with the superior dependency of these cells on ALK signaling. Moreover, when crizotinib and TAE684 were administered for shorter time intervals, all RMS cell lines exhibited comparable cell cycle arrest (at G1 or G2/M phase) and apoptosis (PARP cleavage), which correlated with downregulation of PI3K-AKT (pAKT) and MAPK (pERK) survival signaling pathways (Fig 5A and 5B). In line with these observations, constitutive ligand-independent activation of amplified c-Met receptor is used as molecular marker of susceptibility to tyrosine kinase inhibitors in human gastric cancer cell lines, since only cells exhibiting high-level expression of wild-type c-Met appear to be sensitive to Met inhibition by drug treatment [57,58].

### Multitargeted activity of ALK inhibitors in RMS cells

**Fig 6:**
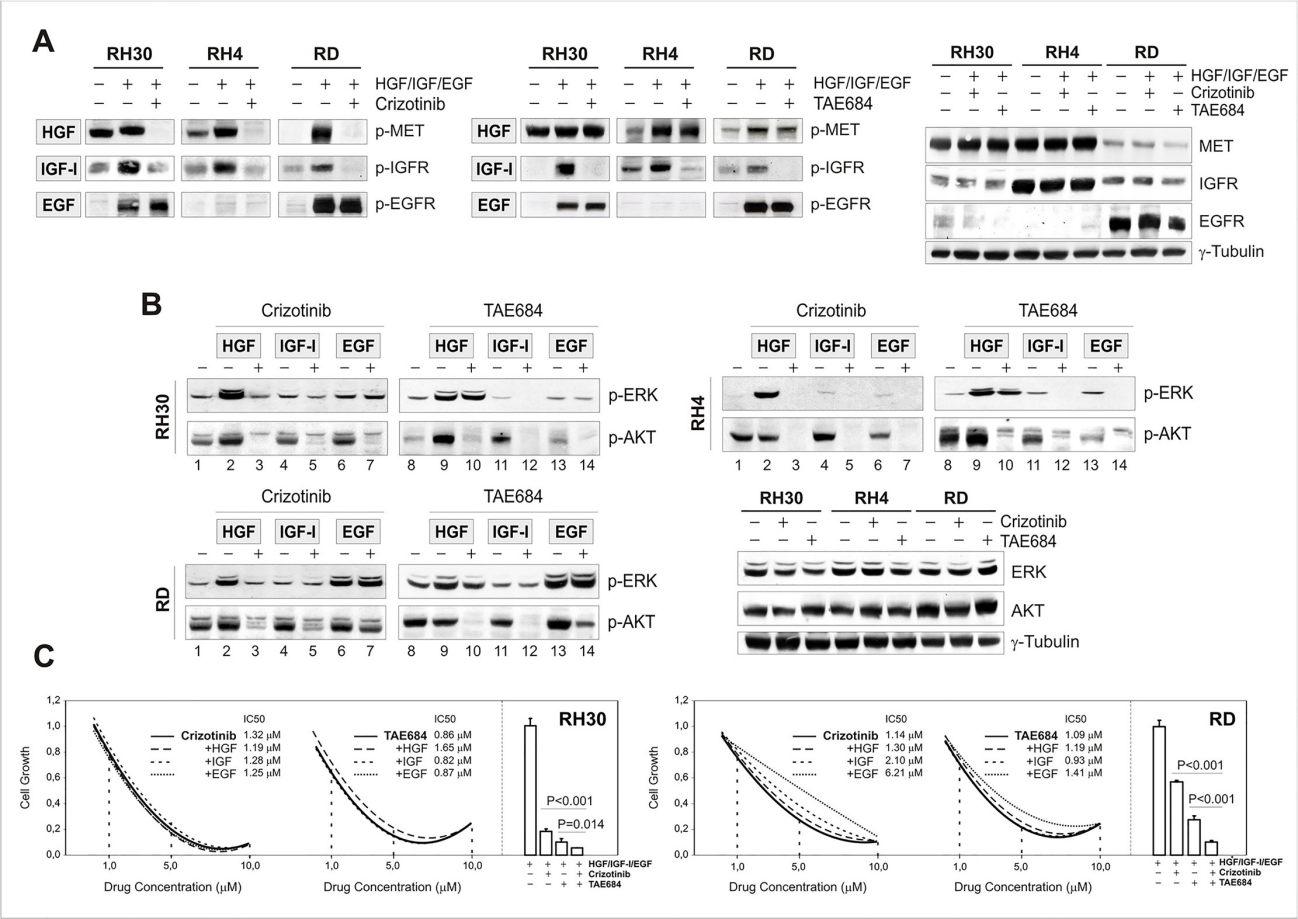
Multitarget inhibitory activity of crizotinib and TAE684 in RMS cells. (A) Western blot analysis of c-Met, IGF-1R and EGFR ligand-dependent phosphorylation. RMS cells were exposed to cognate receptor ligands (50 ng/ml HGF; 200 ng/ml IGF-I; 50 ng/ml EGF), in the presence and absence of Crizotinib or TAE684 inhibitors. Lysates were probed with antibodies directed against the specified proteins and γ-Tubulin. (B) Western blot analysis of c-Met, IGF-1R and EGFR ligand-dependent signalling. Effects on ERK and AKT phosphorylation were assessed using the specified antibodies. γ-Tubulin was used as loading control. (C) Growth inhibition of RH30 and RD cells after 24H-drug treatment, in the presence or absence of HGF, IGF-I or EGF growth factors. Growth curves show average values and IC50 values of triplicates from one representative experiment out of two, whereas bar graphs aside represent combined exposure to HGF, IGF-1 and EGF growth factors in the presence or absence of 5 μM Crizotinib, TAE684 or both for 24 hours.

Cancer cells typically express multiple RTKs that mediate signals through common downstream survival factors, and can be rescued from drug sensitivity by simply exposing them to one or more receptor ligands [59,60,61]. Therefore, to investigate the possibility that potency of ALK inhibitors in RMS cells might be the result of inhibition of other targets, c-MET, IGF-1R and EGFR RTKs were stimulated by cognate ligands and drug-induced perturbation of cell signaling was assessed. Similar to ALK, basal phosphorylation of these receptors was variable in the three RMS cell lines, and in some cases too weak to be discernable without growth factor stimulation. Analysis of c-Met, IGF-1R and EGFR phosphorylation, however, confirmed their activation by cognate ligands (HGF, IGF-I and EGF, respectively) and their expected sensitivity to ALK inhibitors. In particular, HGF stimulated c-Met receptor in the presence of TAE684 but not of crizotinib, IGF-I-dependent activation of IGF-1R was sensitive to both drugs, whereas activated EGFR was resistant either to Crizotinib or TAE684 (Fig 6A). With respect to cell signaling, HGF administration activated ERK (pERK) in all 3 cell lines, whereas EGF stimulated ERK phosphorylation mainly in RD cells (Fig 6B). IGF-I, in contrast, failed to generate additional signaling output despite IGF-1R receptor activation. Moreover, consistent with kinase inhibitors specificity, HGF activated ERK in the presence of TAE684 only (Fig 6B, lanes 2–3 and 9–10), EGF induced ERK phosphorylation in the presence of both inhibitors (Fig 6B, lanes 6–7 and 13–14), whereas IGF was unable to sustain cell signaling during drug treatment (Fig 6B, lanes 4–5 and 11–12). None of these factors, however, were able to completely rescue RMS cells from signaling downregulation, as shown by the almost complete inhibition of AKT phosphorylation in these conditions. Ligand exposure, in fact, had a limited effect on growth and survival of RMS cells, since, except in RD cells exposed to EGF, growth inhibition progressed independently of receptor activation (Fig 6C), and even increased when drug treatments were combined (Fig 6C, *bar graphs*).

## Discussion

In the last few years the rapid development and introduction of targeted agents into clinical practice has reshaped the landscape of cancer therapy and generated a lot of expectations in terms of curability and survival. Nowadays the possibility to identify the most appropriate therapy for a patient is a reality, thanks to the effort put on the identification of potentially druggable targets from the alterations and mutations that characterize cancer cells. Among many factors known to increase the risk of developing cancer, RTKs are of the utmost importance, since when aberrantly expressed they can initiate a variety of signalling pathways that ultimately lead to cell proliferation and survival [62]. Targeting this class of proteins is feasible and a suitable approach to cure cancer. However, the success of this type of treatment clearly depends on the selection of appropriate tumour types whose survival relies on these signalling molecules and patient populations that express functional molecular markers. Indeed, clinical experience has shown that only a percentage of patients respond to targeted therapies even if they express the altered target. Most of tumours treated with tyrosine kinase inhibitors become resistant to treatment due to genetic modification of the primary intended target, gene amplification or constitutive activation of alternative RTKs, but in some cases treatment failure is a consequence of the apparent dispensable nature of the target for cancer growth and survival. Recognizing the difference between a pharmacological inadequacy and the lack of oncogene addiction is, thus, crucial to decide what might constitute a worthy therapeutic target.

In this context, we evaluated ALK receptor expression and activity in RMS cells, to provide a foundation for the use of ALK inhibitors in this malignancy. In rhabdomyosarcoma, a childhood cancer with a very dismal prognosis at advanced stage, multiple RTKs are expressed and regulated by several different transcriptional and post-transcriptional mechanisms. Alterations in gene expression, including amplification and second-site mutations have been described for FGFR-4 and PDGFR receptors, whereas increased autocrine/paracrine growth factor production is responsible for c-Met and IGF-1R signalling [63,64,65,66,67]. In RMS RTKs are considered critical drug targets, but a unique cancer-causing kinase have not been identified yet and RTKs signalling networks appear to be highly intermingled. Consistent with these findings, we found that ALK is mainly expressed in fusion-positive alveolar RMS cells, but, although it localizes at the plasma membrane, basal phosphorylation and intrinsic kinase activity are very low. Such a proper localization coupled to a weak basal activity suggests a role of endogenous phosphatases in maintaining membrane-bound ALK permanently dephosphorylated, but also provides evidences of its potential activation upon growth factor binding [39,43,68,69,70]. Indeed, imbalanced ALK/PTPase activities by either ligand binding or phosphatase inactivation was able to induce ALK phosphorylation and signalling in RMS cells, whereas small-molecule inhibitors or growth factor antagonists exerted opposite effects. Of note, receptor density at the plasma membrane was a determining factor for ALK kinase activity in these cells, even in the presence of counteracting PTPases. ALK overexpression, as that obtained upon transient transfection, resulted, in fact, in a strong up-regulation of receptor autophosphorylation and activity, similar to that seen in ALK-amplified NB1 cells. These findings indicated the level of expression, rather than the presence of activating mutations, as the primary mediator of ALK function in RMS cells, but raised questions about the rationale of targeting a protein whose endogenous expression is not sufficient to sustain constitutive kinase activity and continuous signalling. Indeed, cancer cells where genomic alterations of *ALK* lead to expression of truncated variants, such as NSCLC (EML4-ALK) and ALCL (NPM-ALK), are addicted to ALK signalling because translocations provide dimerization domains that render fusion proteins ligand independent. Tumours expressing full-length ALK, instead, rely on both ligand availability and adequate receptor density to promote homo-dimerization and stimulate activity at the cell surface [71,72,73]. In these cases ALK expression is often of uncertain pathogenic significance and tumour cells do not exhibit the same dependency on receptor signalling. This might explain why ALK targeted inhibitors have shown impressive clinical efficacy in ALK-rearranged malignancies, whereas in cancer patients expressing full-length ALK kinase clinical response remains unsatisfactory [38,74,75,76]. This notion is supported by the observations that in gastric cancers, as well as in a smaller subset of lung and esophageal cancers, sensitivity to c-Met small-molecule inhibitors, including crizotinib, is strongly linked to *Met* gene amplification or receptor overexpression, as in EGFR-positive NSCLC tumours responsiveness to kinase inhibitors correlates with both *EGFR* and *HER2* gene amplification [77,78,79].

Cancer cells, however, are know for their exquisite capacity to survive in many adverse conditions through the expression and activity of different oncogenic drivers, including several RTKs. In these circumstances, tyrosine kinase inhibitors, despite being optimized against a specific protein kinase, may be used for their potential to bind and inhibit multiple alternative targets, motivating their subsequent *in vitro* and *in vivo* investigation [53]. Indeed, in accordance with previously reported data [80], we found that crizotinib and TAE684 kinase inhibitors were active on RMS cells independently of ALK expression, most likely due to their group-selective nature. We verified this by assessing baseline and ligand-induced phosphorylation of few important RTKs expressed in RMS cells, before and after drug treatment. Targeting constitutively active or ligand-induced c-Met, IGF-1R and EGFR receptors with either crizotinib or TAE684 was demonstrated, and although in some cases cognate ligands were able to partially rescue cells from single-agent treatment (EGF), concurrent administration of both compounds prevented this effect and improved treatment efficacy. In line with our observations, Ponatinib, a small-molecule FGFR4 inhibitor initially developed for native and mutant BCR-ABL kinase, is active on RMS cells with different FGFR4 expression levels, due, perhaps, to its multi-target inhibitor activity [81]. Similar, crizotinib can be administered to patients with ROS1-rearranged NSCLC that do not express ALK or c-Met, as LDK378 (Ceritinib) and AP26113 ALK inhibitors demonstrate clinical efficacy on crizotinib-resistant NSCLC tumours with increased abundance of IGF-1R and ROS proteins [82,83,84].

## Conclusions

Collectively, our observations suggest that ALK may represent a potentially druggable target in RMS, although remains to be clarified whether RMS may be considered an ALK-positive tumour or an ALK-driven malignancy. Most importantly, we provided evidence that assessment of ALK kinase activity, or simply its phosphorylation status, constitutes an important molecular marker for drug response *in vivo* and should be properly evaluated when proposing this kinase for targeted therapy. In this context, RMS tumours that overexpress ALK may constitute a subset at high likelihood for drug response, whereas those harbouring mutations in the kinase domain or expressing wild-type ALK receptor at physiological levels may not be sensitive to targeted treatments. Selection of RMS tumours genotyped in early-phase clinical trials for gene amplification or assessed for constitutive kinase activity may allow demonstration of specific drug effectiveness while limiting patient cohorts size. In contrast, combinatorial treatments or multitargeted kinase inhibitors may be of most efficacy in those cases where non-functional ALK is compensated by parallel expression of alternative RTKs. In both cases, however, combination of genomic, quantitative transcriptomics and proteomics data with functional screens will certainly help to design better therapies that prevent and overcome resistance to treatment in RMS cancer patients.

## References

[1] Lemmon MA, Schlessinger J (2010) Cell signaling by receptor tyrosine kinases. Cell 141: 1117–1134. 10.1016/j.cell.2010.06.011 20602996 PMC2914105

[2] Sun K, Wang ZM, Xu LJ, Teng XD, Yao HT, et al (2007) [Inflammatory myofibroblastic tumor of bladder: a clinicopathologic study of five cases]. Zhonghua Bing Li Xue Za Zhi 36: 605–608. 18070449

[3] Druker BJ, Talpaz M, Resta DJ, Peng B, Buchdunger E, Ford JM et al (2001) Efficacy and safety of a specific inhibitor of the BCR-ABL tyrosine kinase in chronic myeloid leukemia. N Engl J Med 344: 1031–1037. 11287972 10.1056/NEJM200104053441401

[4] Flaherty KT, Puzanov I, Kim KB, Ribas A, McArthur GA, Sosman JA et al (2010) Inhibition of mutated, activated BRAF in metastatic melanoma. N Engl J Med 363: 809–819. 10.1056/NEJMoa1002011 20818844 PMC3724529

[5] Heinrich MC, Corless CL, Demetri GD, Blanke CD, von Mehren M, Joensuu H et al (2003) Kinase mutations and imatinib response in patients with metastatic gastrointestinal stromal tumor. J Clin Oncol 21: 4342–4349. 14645423 10.1200/JCO.2003.04.190

[6] Lynch TJ, Bell DW, Sordella R, Gurubhagavatula S, Okimoto RA, Branningan BW et al (2004) Activating mutations in the epidermal growth factor receptor underlying responsiveness of non-small-cell lung cancer to gefitinib. N Engl J Med 350: 2129–2139. 15118073 10.1056/NEJMoa040938

[7] Niederst MJ, Engelman JA (2013) Bypass mechanisms of resistance to receptor tyrosine kinase inhibition in lung cancer. Sci Signal 6: re6. 10.1126/scisignal.2004652 PMC3876281 24065147

[8] Engelman JA, Zejnullahu K, Mitsudomi T, Song Y, Hyland C, Park JO et al (2007) MET amplification leads to gefitinib resistance in lung cancer by activating ERBB3 signaling. Science 316: 1039–1043. 17463250 10.1126/science.1141478

[9] Poulikakos PI, Persaud Y, Janakiraman M, Kong X, Ng C, Moiceau G et al (2011) RAF inhibitor resistance is mediated by dimerization of aberrantly spliced BRAF(V600E). Nature 480: 387–390. 10.1038/nature10662 22113612 PMC3266695

[10] Jackman D, Pao W, Riely GJ, Engelman JA, Kris MG, Janne PA et al (2010) Clinical definition of acquired resistance to epidermal growth factor receptor tyrosine kinase inhibitors in non-small-cell lung cancer. J Clin Oncol 28: 357–360. 10.1200/JCO.2009.24.7049 19949011 PMC3870288

[11] Katayama R, Shaw AT, Khan TM, Mino-Kenudson M, Solomon BJ, Halmos B et al (2012) Mechanisms of acquired crizotinib resistance in ALK-rearranged lung Cancers. Sci Transl Med 4: 120ra117. 10.1126/scitranslmed.3003316 PMC3385512 22277784

[12] Nazarian R, Shi H, Wang Q, Kong X, Koya RC, Lee H et al (2010) Melanomas acquire resistance to B-RAF(V600E) inhibition by RTK or N-RAS upregulation. Nature 468: 973–977. 10.1038/nature09626 21107323 PMC3143360

[13] Prahallad A, Sun C, Huang S, Di Nicolantonio F, Salazar R, Zecchin D et al (2012) Unresponsiveness of colon cancer to BRAF(V600E) inhibition through feedback activation of EGFR. Nature 483: 100–103. 10.1038/nature10868 22281684

[14] Cao L, Yu Y, Bilke S, Walker RL, Mayeenuddin LH, Azorsa DO et al (2010) Genome-wide identification of PAX3-FKHR binding sites in rhabdomyosarcoma reveals candidate target genes important for development and cancer. Cancer Res 70: 6497–6508. 10.1158/0008-5472.CAN-10-0582 20663909 PMC2922412

[15] Crose LE, Linardic CM (2011) Receptor tyrosine kinases as therapeutic targets in rhabdomyosarcoma. Sarcoma: 756982. 10.1155/2011/756982 PMC3022188 21253475

[16] Davicioni E, Finckenstein FG, Shahbazian V, Buckley JD, Triche TJ, Anderson MJ (2006) Identification of a PAX-FKHR gene expression signature that defines molecular classes and determines the prognosis of alveolar rhabdomyosarcomas. Cancer Res 66: 6936–6946. 16849537 10.1158/0008-5472.CAN-05-4578

[17] Armistead PM, Salganick J, Roh JS, Steinert DM, Patel S, Munsell M et al (2007) Expression of receptor tyrosine kinases and apoptotic molecules in rhabdomyosarcoma: correlation with overall survival in 105 patients. Cancer 110: 2293–2303. 17896786 10.1002/cncr.23038

[18] Ganti R, Skapek SX, Zhang J, Fuller CE, Wu J, Billups CA et al (2006) Expression and genomic status of EGFR and ErbB-2 in alveolar and embryonal rhabdomyosarcoma. Mod Pathol 19: 1213–1220. 16729016 10.1038/modpathol.3800636

[19] Rees H, Williamson D, Papanastasiou A, Jina N, Nabarro S, Shipley J et al (2006) The MET receptor tyrosine kinase contributes to invasive tumour growth in rhabdomyosarcomas. Growth Factors 24: 197–208. 17079203 10.1080/08977190600759923

[20] Yeung CL, Ngo VN, Grohar PJ, Arnaldez FI, Asante A, Wan X et al (2013) Loss-of-function screen in rhabdomyosarcoma identifies CRKL-YES as a critical signal for tumor growth. Oncogene 32: 5429–5438. 10.1038/onc.2012.590 23318429 PMC3898328

[21] Diomedi-Camassei F, McDowell HP, De Ioris MA, Uccini S, Altavista P, Rachella G et al (2008) Clinical significance of CXC chemokine receptor-4 and c-Met in childhood rhabdomyosarcoma. Clin Cancer Res 14: 4119–4127. 10.1158/1078-0432.CCR-07-4446 18593989

[22] Huang F, Hurlburt W, Greer A, Reeves KA, Hillerman S, Chang H et al (2010) Differential mechanisms of acquired resistance to insulin-like growth factor-i receptor antibody therapy or to a small-molecule inhibitor, BMS-754807, in a human rhabdomyosarcoma model. Cancer Res 70: 7221–7231. 10.1158/0008-5472.CAN-10-0391 20807811

[23] Lukasiewicz E, Miekus K, Kijowski J, Drabik G, Wilusz M, Bobis-Wozowicz S et al (2009) Inhibition of rhabdomyosarcoma's metastatic behavior through downregulation of MET receptor signaling. Folia Histochem Cytobiol 47: 485–489. 10.2478/v10042-009-0108-x 20164036

[24] Taulli R, Scuoppo C, Bersani F, Accornero P, Forni PE, Miretti S et al (2006) Validation of met as a therapeutic target in alveolar and embryonal rhabdomyosarcoma. Cancer Res 66: 4742–4749. 16651427 10.1158/0008-5472.CAN-05-4292

[25] Bonvini P, Zin A, Alaggio R, Pawel B, Bisogno G, Rosolen A (2013) High ALK mRNA expression has a negative prognostic significance in rhabdomyosarcoma. Br J Cancer 109: 3084–3091. 10.1038/bjc.2013.653 24149177 PMC3859940

[26] van Gaal JC, Flucke UE, Roeffen MH, de Bont ES, Sleijfer S, Mavinkurve-Groothuis AM et al (2013) Anaplastic lymphoma kinase aberrations in rhabdomyosarcoma: clinical and prognostic implications. J Clin Oncol 30: 308–315. 10.1200/JCO.2011.37.8588 22184391

[27] Yoshida A, Shibata T, Wakai S, Ushiku T, Tsuta K, Fukayama M et al. (2013) Anaplastic lymphoma kinase status in rhabdomyosarcomas. Mod Pathol. 10.1038/modpathol.2012.222 23307059

[28] Cessna MH, Zhou H, Sanger WG, Perkins SL, Tripp S, Pickering D et al (2002) Expression of ALK1 and p80 in inflammatory myofibroblastic tumor and its mesenchymal mimics: a study of 135 cases. Mod Pathol 15: 931–938. 12218210 10.1097/01.MP.0000026615.04130.1F

[29] Chen Y, Takita J, Choi YL, Kato M, Ohira M, Sanada M et al (2008) Oncogenic mutations of ALK kinase in neuroblastoma. Nature 455: 971–974. 10.1038/nature07399 18923524

[30] Lawrence B, Perez-Atayde A, Hibbard MK, Rubin BP, Dal Cin P, Pinkus JL et al (2000) TPM3-ALK and TPM4-ALK oncogenes in inflammatory myofibroblastic tumors. Am J Pathol 157: 377–384. 10934142 10.1016/S0002-9440(10)64550-6 PMC1850130

[31] Pulford K, Lamant L, Morris SW, Butler LH, Wood KM, Stroud D et al (1997) Detection of anaplastic lymphoma kinase (ALK) and nucleolar protein nucleophosmin (NPM)-ALK proteins in normal and neoplastic cells with the monoclonal antibody ALK1. Blood 89: 1394–1404. 9028963

[32] Soda M, Choi YL, Enomoto M, Takada S, Yamashita Y, Ishikawa S et al (2007) Identification of the transforming EML4-ALK fusion gene in non-small-cell lung cancer. Nature 448: 561–566. 17625570 10.1038/nature05945

[33] Bresler SC, Weiser DA, Huwe PJ, Park JH, Krytska K, Ryles H et al (2014) ALK mutations confer differential oncogenic activation and sensitivity to ALK inhibition therapy in neuroblastoma. Cancer Cell 26: 682–694. 10.1016/j.ccell.2014.09.019 25517749 PMC4269829

[34] Christensen JG, Zou HY, Arango ME, Li Q, Lee JH, McDonnell SR et al (2007) Cytoreductive antitumor activity of PF-2341066, a novel inhibitor of anaplastic lymphoma kinase and c-Met, in experimental models of anaplastic large-cell lymphoma. Mol Cancer Ther 6: 3314–3322. 18089725 10.1158/1535-7163.MCT-07-0365

[35] Iwama E, Okamoto I, Harada T, Takayama K, Nakanishi Y (2014) Development of anaplastic lymphoma kinase (ALK) inhibitors and molecular diagnosis in ALK rearrangement-positive lung cancer. Onco Targets Ther 7: 375–385. 10.2147/OTT.S38868 24623980 PMC3949762

[36] Shaw AT, Solomon B (2011) Targeting anaplastic lymphoma kinase in lung cancer. Clin Cancer Res 17: 2081–2086. 10.1158/1078-0432.CCR-10-1591 21288922

[37] Kwak EL, Bang YJ, Camidge DR, Shaw AT, Solomon B, Branningan BW et al (2010) Anaplastic lymphoma kinase inhibition in non-small-cell lung cancer. N Engl J Med 363: 1693–1703. 10.1056/NEJMoa1006448 20979469 PMC3014291

[38] Mosse YP, Lim MS, Voss SD, Wilner K, Ruffner K, Laliberte J et al (2013) Safety and activity of crizotinib for paediatric patients with refractory solid tumours or anaplastic large-cell lymphoma: a Children's Oncology Group phase 1 consortium study. Lancet Oncol 14: 472–480. 10.1016/S1470-2045(13)70095-0 23598171 PMC3730818

[39] Moog-Lutz C, Degoutin J, Gouzi JY, Frobert Y, Brunet-de Carvalho N, Bureau J et al (2005) Activation and inhibition of anaplastic lymphoma kinase receptor tyrosine kinase by monoclonal antibodies and absence of agonist activity of pleiotrophin. J Biol Chem 280: 26039–26048. 15886198 10.1074/jbc.M501972200

[40] Huyer G, Liu S, Kelly J, Moffat J, Payette P, Kennedy B et al (1997) Mechanism of inhibition of protein-tyrosine phosphatases by vanadate and pervanadate. J Biol Chem 272: 843–851. 8995372 10.1074/jbc.272.2.843

[41] Bonvini P, Gastaldi T, Falini B, Rosolen A (2002) Nucleophosmin-anaplastic lymphoma kinase (NPM-ALK), a novel Hsp90-client tyrosine kinase: down-regulation of NPM-ALK expression and tyrosine phosphorylation in ALK(+) CD30(+) lymphoma cells by the Hsp90 antagonist 17-allylamino,17-demethoxygeldanamycin. Cancer Res 62: 1559–1566. 11888936

[42] Mathivet T, Mazot P, Vigny M (2007) In contrast to agonist monoclonal antibodies, both C-terminal truncated form and full length form of Pleiotrophin failed to activate vertebrate ALK (anaplastic lymphoma kinase)? Cell Signal 19: 2434–2443. 17904822 10.1016/j.cellsig.2007.07.011

[43] Mazot P, Cazes A, Boutterin MC, Figueiredo A, Raynal V, Combaret V et al (2011) The constitutive activity of the ALK mutated at positions F1174 or R1275 impairs receptor trafficking. Oncogene 30: 2017–2025. 10.1038/onc.2010.595 21242967

[44] Volinsky N, Kholodenko BN (2013) Complexity of receptor tyrosine kinase signal processing. Cold Spring Harb Perspect Biol 5: a009043 10.1101/cshperspect.a009043 23906711 PMC3721286

[45] Casaletto JB, McClatchey AI (2012) Spatial regulation of receptor tyrosine kinases in development and cancer. Nat Rev Cancer 12: 387–400. 10.1038/nrc3277 22622641 PMC3767127

[46] Clayton AH, Tavarnesi ML, Johns TG (2007) Unligated epidermal growth factor receptor forms higher order oligomers within microclusters on A431 cells that are sensitive to tyrosine kinase inhibitor binding. Biochemistry 46: 4589–4597. 17381163 10.1021/bi700002b

[47] Ward CW, Lawrence MC, Streltsov VA, Adams TE, McKern NM (2007) The insulin and EGF receptor structures: new insights into ligand-induced receptor activation. Trends Biochem Sci 32: 129–137. 17280834 10.1016/j.tibs.2007.01.001

[48] Janoueix-Lerosey I, Lequin D, Brugieres L, Ribeiro A, de Pontual L, Combaret V et al (2008) Somatic and germline activating mutations of the ALK kinase receptor in neuroblastoma. Nature 455: 967–970. 10.1038/nature07398 18923523

[49] Mazot P, Cazes A, Dingli F, Degoutin J, Irinopoulou T, Boutterin MC et al (2012) Internalization and down-regulation of the ALK receptor in neuroblastoma cell lines upon monoclonal antibodies treatment. PLoS One 7: e33581 10.1371/journal.pone.0033581 22479414 PMC3316580

[50] Ostman A, Bohmer FD (2001) Regulation of receptor tyrosine kinase signaling by protein tyrosine phosphatases. Trends Cell Biol 11: 258–266. 11356362 10.1016/s0962-8924(01)01990-0

[51] Chung I, Akita R, Vandlen R, Toomre D, Schlessinger J, Mellman I (2010) Spatial control of EGF receptor activation by reversible dimerization on living cells. Nature 464: 783–787. 10.1038/nature08827 20208517

[52] Knight JD, Qian B, Baker D, Kothary R (2007) Conservation, variability and the modeling of active protein kinases. PLoS One 2: e982 17912359 10.1371/journal.pone.0000982 PMC1989141

[53] Davis MI, Hunt JP, Herrgard S, Ciceri P, Wodicka LM, Pallares G et al (2011) Comprehensive analysis of kinase inhibitor selectivity. Nat Biotechnol 29: 1046–1051. 10.1038/nbt.1990 22037378

[54] Wagner JP, Wolf-Yadlin A, Sevecka M, Grenier JK, Root DE, Lauffenburger DA et al (2013) Receptor tyrosine kinases fall into distinct classes based on their inferred signaling networks. Sci Signal 6: ra58. 10.1126/scisignal.2003994 PMC3987808 23861540

[55] Galkin AV, Melnick JS, Kim S, Hood TL, Li N, Li L et al (2007) Identification of NVP-TAE684, a potent, selective, and efficacious inhibitor of NPM-ALK. Proc Natl Acad Sci U S A 104: 270–275. 17185414 10.1073/pnas.0609412103 PMC1765448

[56] Rodig SJ, Shapiro GI (2010) Crizotinib, a small-molecule dual inhibitor of the c-Met and ALK receptor tyrosine kinases. Curr Opin Investig Drugs 11: 1477–1490. 21154129

[57] McDermott U, Sharma SV, Dowell L, Greninger P, Montagut C, Lamb J et al (2007) Identification of genotype-correlated sensitivity to selective kinase inhibitors by using high-throughput tumor cell line profiling. Proc Natl Acad Sci U S A 104: 19936–19941. 18077425 10.1073/pnas.0707498104 PMC2148401

[58] Smolen GA, Sordella R, Muir B, Mohapatra G, Barmettler A, Archibald H et al (2006) Amplification of MET may identify a subset of cancers with extreme sensitivity to the selective tyrosine kinase inhibitor PHA-665752. Proc Natl Acad Sci U S A 103: 2316–2321. 16461907 10.1073/pnas.0508776103 PMC1413705

[59] Wilson TR, Fridlyand J, Yan Y, Penuel E, Burton L, Chan E et al (2012) Widespread potential for growth-factor-driven resistance to anticancer kinase inhibitors. Nature 487: 505–509. 10.1038/nature11249 22763448 PMC3724525

[60] Yamada T, Takeuchi S, Nakade J, Kita K, Nakagawa T, Nanjo S et al (2012) Paracrine receptor activation by microenvironment triggers bypass survival signals and ALK inhibitor resistance in EML4-ALK lung cancer cells. Clin Cancer Res 18: 3592–3602. 10.1158/1078-0432.CCR-11-2972 22553343

[61] Yano S, Takeuchi S, Nakagawa T, Yamada T (2012) Ligand-triggered resistance to molecular targeted drugs in lung cancer: roles of hepatocyte growth factor and epidermal growth factor receptor ligands. Cancer Sci 103: 1189–1194. 10.1111/j.1349-7006.2012.02279.x 22435662 PMC7659280

[62] Schlessinger J (2014) Receptor tyrosine kinases: legacy of the first two decades. Cold Spring Harb Perspect Biol 6. 10.1101/cshperspect.a008912 PMC3949355 24591517

[63] Anastasi S, Giordano S, Sthandier O, Gambarotta G, Maione R, Comoglio P et al (1997) A natural hepatocyte growth factor/scatter factor autocrine loop in myoblast cells and the effect of the constitutive Met kinase activation on myogenic differentiation. J Cell Biol 137: 1057–1068. 9166406 10.1083/jcb.137.5.1057 PMC2136220

[64] Ehnman M, Missiaglia E, Folestad E, Selfe J, Strell C, Thway K et al (2013) Distinct effects of ligand-induced PDGFRalpha and PDGFRbeta signaling in the human rhabdomyosarcoma tumor cell and stroma cell compartments. Cancer Res 73: 2139–2149. 10.1158/0008-5472.CAN-12-1646 23338608 PMC3672973

[65] El-Badry OM, Minniti C, Kohn EC, Houghton PJ, Daughaday WH, Helman LJ (1990) Insulin-like growth factor II acts as an autocrine growth and motility factor in human rhabdomyosarcoma tumors. Cell Growth Differ 1: 325–331. 2177632

[66] Shern JF, Chen L, Chmielecki J, Wei JS, Patidar R, Rosenberg M et al (2014) Comprehensive genomic analysis of rhabdomyosarcoma reveals a landscape of alterations affecting a common genetic axis in fusion-positive and fusion-negative tumors. Cancer Discov 4: 216–231. 10.1158/2159-8290.CD-13-0639 24436047 PMC4462130

[67] Taylor JGt, Cheuk AT, Tsang PS, Chung JY, Song YK, Desai K et al (2009) Identification of FGFR4-activating mutations in human rhabdomyosarcomas that promote metastasis in xenotransplanted models. J Clin Invest 119: 3395–3407. 10.1172/JCI39703 19809159 PMC2769177

[68] Boutterin MC, Mazot P, Faure C, Doly S, Gervasi N, Tremblay ML et al (2013) Control of ALK (wild type and mutated forms) phosphorylation: specific role of the phosphatase PTP1B. Cell Signal 25: 1505–1513. 10.1016/j.cellsig.2013.02.020 23499906

[69] Motegi A, Fujimoto J, Kotani M, Sakuraba H, Yamamoto T (2004) ALK receptor tyrosine kinase promotes cell growth and neurite outgrowth. J Cell Sci 117: 3319–3329. 15226403 10.1242/jcs.01183

[70] Perez-Pinera P, Zhang W, Chang Y, Vega JA, Deuel TF (2007) Anaplastic lymphoma kinase is activated through the pleiotrophin/receptor protein-tyrosine phosphatase beta/zeta signaling pathway: an alternative mechanism of receptor tyrosine kinase activation. J Biol Chem 282: 28683–28690. 17681947 10.1074/jbc.M704505200

[71] Friboulet L, Li N, Katayama R, Lee CC, Gainor JF, Crystal AS et al (2014) The ALK inhibitor ceritinib overcomes crizotinib resistance in non-small cell lung cancer. Cancer Discov 4: 662–673. 10.1158/2159-8290.CD-13-0846 24675041 PMC4068971

[72] Lovly CM, Heuckmann JM, de Stanchina E, Chen H, Thomas RK, Liang C et al (2011) Insights into ALK-driven cancers revealed through development of novel ALK tyrosine kinase inhibitors. Cancer Res 71: 4920–4931. 10.1158/0008-5472.CAN-10-3879 21613408 PMC3138877

[73] McDermott U, Iafrate AJ, Gray NS, Shioda T, Classon M, Maheswaran S et al (2008) Genomic alterations of anaplastic lymphoma kinase may sensitize tumors to anaplastic lymphoma kinase inhibitors. Cancer Res 68: 3389–3395. 10.1158/0008-5472.CAN-07-6186 18451166

[74] Camidge DR, Bang YJ, Kwak EL, Iafrate AJ, Varella-Garcia M, Fox SB et al (2012) Activity and safety of crizotinib in patients with ALK-positive non-small-cell lung cancer: updated results from a phase 1 study. Lancet Oncol 13: 1011–1019. 10.1016/S1470-2045(12)70344-3 22954507 PMC3936578

[75] Seto T, Kiura K, Nishio M, Nakagawa K, Maemondo M, Inoue A et al (2013) CH5424802 (RO5424802) for patients with ALK-rearranged advanced non-small-cell lung cancer (AF-001JP study): a single-arm, open-label, phase 1–2 study. Lancet Oncol 14: 590–598. 10.1016/S1470-2045(13)70142-6 23639470

[76] Shaw AT, Engelman JA (2014) Ceritinib in ALK-rearranged non-small-cell lung cancer. N Engl J Med 370: 2537–2539. 10.1056/NEJMc1404894 24963575

[77] Lennerz JK, Kwak EL, Ackerman A, Michael M, Fox SB, Bergethon K et al (2011) MET amplification identifies a small and aggressive subgroup of esophagogastric adenocarcinoma with evidence of responsiveness to crizotinib. J Clin Oncol 29: 4803–4810. 10.1200/JCO.2011.35.4928 22042947 PMC3255989

[78] Okamoto W, Okamoto I, Arao T, Kuwata K, Hatashita E, Yamaguchi H et al (2012) Antitumor action of the MET tyrosine kinase inhibitor crizotinib (PF-02341066) in gastric cancer positive for MET amplification. Mol Cancer Ther 11: 1557–1564. 10.1158/1535-7163.MCT-11-0934 22729845

[79] Zou HY, Li Q, Lee JH, Arango ME, Burgess K, Qiu M et al (2012) Sensitivity of selected human tumor models to PF-04217903, a novel selective c-Met kinase inhibitor. Mol Cancer Ther 11: 1036–1047. 10.1158/1535-7163.MCT-11-0839 22389468

[80] Nishimura R, Takita J, Sato-Otsubo A, Kato M, Koh K, Hanada R et al (2013) Characterization of genetic lesions in rhabdomyosarcoma using a high-density single nucleotide polymorphism array. Cancer Sci 104: 856–864. 10.1111/cas.12173 23578105 PMC7657110

[81] Li SQ, Cheuk AT, Shern JF, Song YK, Hurd L, Liao H et al (2013) Targeting wild-type and mutationally activated FGFR4 in rhabdomyosarcoma with the inhibitor ponatinib (AP24534). PLoS One 8: e76551 10.1371/journal.pone.0076551 24124571 PMC3790700

[82] Chen J, Jiang C Wang S LDK378: a promising anaplastic lymphoma kinase (ALK) inhibitor. J Med Chem 56: 5673–5674. 10.1021/jm401005u 23837797

[83] Lovly CM, McDonald NT, Chen H, Ortiz-Cuaran S, Heukamp LC, Yan Y et al (2014) Rationale for co-targeting IGF-1R and ALK in ALK fusion-positive lung cancer. Nat Med 20: 1027–1034. 10.1038/nm.3667 25173427 PMC4159407

[84] Sabbatini P, Korenchuk S, Rowand JL, Groy A, Liu Q, Leperi D et al (2009) GSK1838705A inhibits the insulin-like growth factor-1 receptor and anaplastic lymphoma kinase and shows antitumor activity in experimental models of human cancers. Mol Cancer Ther 8: 2811–2820. 10.1158/1535-7163.MCT-09-0423 19825801

